# Total Arch Replacement is Safe as Hemiarch Repair in Acute Type A Aortic Dissection in a Middle-Income Country Setting

**DOI:** 10.21470/1678-9741-2024-0088

**Published:** 2025-02-05

**Authors:** Juan David Niño-Calvera, Julian Senosiain, Nicolas Nuñez-Ordonez, Ivonne Pineda, Lina Ramírez, Carlos Villa, Carlos Obando, Tomas Chalela, Nestor Sandoval, Juan P Umaña, Jaime Camacho

**Affiliations:** 1 Cardiovascular Surgery Department, Fundación Cardioinfantil – Instituto de Cardiologia, Bogotá, Colombia

**Keywords:** Surgical Wound Infection, Reoperation, Hospital Mortality, Aortic Dissection, Spinal Cord Injuries, Stroke, Acute Kidney Injury

## Abstract

**Objectiver:**

The aim of this study was to determine the clinical outcomes of patients with acute type A aortic dissection comparing proximal aortic repair *vs.* total arch replacement.

**Methods:**

This was a retrospective cohort study. We included all acute type A aortic dissection procedures from January 2002 to November 2022. Groups were defined according to the extent of aortic replacement (hemiarch repair *vs.* total arch replacement). We collected data from pre, intra, and postoperative variables. Our main endpoints were stroke rate, spinal cord injury, and in-hospital mortality. We performed a statistical analysis for between-group comparisons according to the nature and distribution of variables. Bivariate analyses were done using the Mann-Whitney U test and for categorical variables, the Chi-square test or Fisher’s exact test. Significance was established at alpha level of 0.05.

**Results:**

We identified 107 acute type A aortic dissection procedures (69 hemiarch repairs *vs.* 38 total arch replacements). There were no differences in postoperative outcomes such as surgical site infection or acute kidney injury. Bleeding reoperation was more frequent in the hemiarch group (30% *vs.* 11 %). We did not find statistically significant differences in stroke rate, spinal cord injury, or in-hospital mortality.

**Conclusion:**

Acute type A aortic dissection treatment is still a challenge. Total arch replacement does not increase the risk of major early postoperative complications in comparison to hemiarch repair. The extended repair seems to provide benefits such as a lower risk of reoperation. Total arch replacement should be performed in selected patients, as it may improve long-term outcomes

## INTRODUCTION

Acute type A aortic dissection (ATAAD) is a challenging surgical emergency that requires early diagnosis and optimal surgical decision making. It is the most frequent fatal aortic syndrome with an estimated incidence of 3-5 cases per 100.000 people every year and it might be underestimated due to deaths before admission^[1]^. The incidence is higher in men with the peak within the 6^th^ decade of life^[2,3]^. In large scale series, authors report an in-hospital mortality ranging from 17 to 26% remaining as a challenge for cardiothoracic surgeons^[4]^.

Treatment of choice is the open surgery for most patients. The goal is the resection of the primary entry tear through a graft interposition in the involved aortic segment, thus re-establishing blood flow to the true lumen^[5,6]^. This procedure is effective in preventing fatal complications and death and it may be achieved either by hemiarch or total arch replacement (TAR) depending on the location of the primary entry tear, surgeon preference, and centre experience in open aortic surgery^[6,7]^.

The hemiarch repair is the preferred surgical approach among most groups because it is less technically demanding. TAR, on the other hand, is a more complex procedure that requires longer periods of circulatory arrest, cardiopulmonary bypass (CPB), and greater surgical expertise^[8,9]^. Nevertheless, evidence is beginning to prove effectiveness of TAR by improving complete false lumen thrombosis and distal aortic remodelling, thus preventing long-term adverse outcomes such as aortic dilation, rupture, or new distal entry tears. The rationale for a more aggressive approach (specially in younger patients and those with connective tissue disorders) is to induce complete false lumen thrombosis and aortic remodelling in the long term^[10]^. Besides the increasing of performing TAR procedures, last decade trend is toward the use of the newer hybrid techniques. The frozen elephant trunk (FET), the B-SAFER procedure, and the warm stented techniques have become popular as extended arch techniques with potentially improved long-term outcomes^[11]^.

However, the best surgical procedure to treat patients with ATAAD is still unclear. While some large-scale registries show no difference in terms of mortality, stroke, or paraplegia rates between hemiarch, TAR, and FET, there is also opposing evidence showing worse outcomes with TAR as compared to hemiarch repair^[12,13]^.

Therefore, this study aimed to compare the early clinical outcomes of patients with ATAAD who underwent proximal aortic repair or extended arch repair in a reference centre in Colombia.

## METHODS

**Fig. 1:**
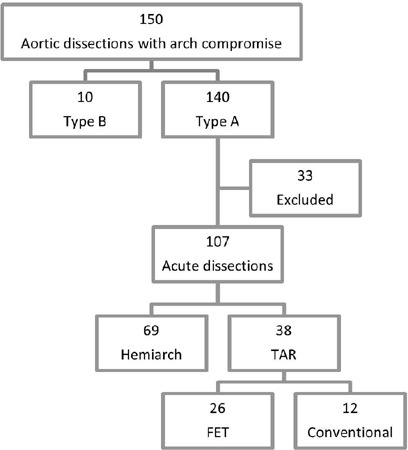
Patient flowchart selection. FET=frozen elephant trunk; TAR=total arch replacement.

This single-centre retrospective cohort study was conducted in a high-complexity hospital in Bogotá, Colombia. Using the electronic registry of the Cardiovascular Surgery Department that follows the guidelines of the Society of Thoracic Surgeons (STS) Adult Cardiac Surgical Database, we identified all aortic dissections with arch compromise between January 2002 and November 2022. Then we included all patients diagnosed with ATAAD who underwent surgical aortic repair within 14 days after symptom onset in this period. Patient flowchart selection is shown in Figure 1. Patients who underwent aortic debranching were excluded from this study, and no other exclusion criteria were considered. We included data from pre, intra, and postoperative variables. Groups of analysis were defined by the extent of the repair that was performed (hemiarch repair vs. TAR). Approval was obtained from our Institutional Review Board (CEIC-0395-2022), and the need for individual informed consent was waived.

The diagnosis of ATAAD was made by computerized tomography in all patients.The decision of the extension of the graft was determined by surgeon’s choice on an individualized evaluation considering the location and extension of the entry tear, the patient’s clinical and hemodynamic status, and comorbidities. All repairs were performed through a median sternotomy using CPB, anterograde cardioplegia, hypothermic circulatory arrest, and anterograde cerebral perfusion. Baseline characteristics included age, sex, comorbidities, history of previous cardiac surgery, left ventricular ejection fraction, risk of in-hospital mortality (estimated by the European System for Cardiac Operative Risk Evaluation II), and functional class (assessed by New York Heart Association [NYHA] functional class classification).

### Definition

Chronicity of aortic dissection was defined following the 2022 American College of Cardiology/American Heart Association guidelines for the diagnosis and management of aortic disease that uses the definition for acute and hyperacute aortic dissection proposed by the Society for Vascular Surgery/STS as following: hyperacute occurring in < 24 hours, and acute developing in a one day- 14 days interval from the onset of symptoms^[14]^. TAR was defined as an aortic repair involving the whole aortic arch including its branches using the conventional elephant trunk or the hybrid FET techniques. The hemiarch repair was defined as an aortic repair limited to the ascending aorta including the lesser curvature of the aortic arch and with no involvement of arch branches.

Surgical priority was divided into three categories following the STS database coding: *urgent* priority as a surgery performed within the same in-hospital stay, *emergency* as critical refractory ill with or without hemodynamic impairment patients who needs immediate cardiac surgery, and *emergent salvage* as patients requiring extracorporeal membrane oxygenation for keeping alive or under cardiac arrest who requires cardiopulmonary resuscitation on the way to the operating room or before the anesthesia induction.

### Outcomes

The primary outcomes of this study were stroke rate, spinal cord injury (SCI), and in-hospital mortality. Stroke rate was defined as any acute focal brain function impairment with imaging evidence of recent brain ischemia occurring during total in-hospital stay after the surgery. SCI was defined as an acute neurological deficit of the spinal cord of new onset after the surgery. Death during hospital or intensive care unit (ICU) stay after surgery was defined as in-hospital mortality.

The secondary endpoints included mediastinum or sternum infection classified as surgical site infection, and acute kidney injury (AKI) defined as any kidney function impairment that accomplished the Kidney Disease Improving Global Outcomes criteria for AKI after surgery. Bleeding reoperation was determined as the needing of a second intervention due to clear signs of internal bleeding after the surgery. All outcomes were measured during the total in-hospital stay follow-up.

### Statistical Analysis

Standard statistical analysis was performed for between-group continuous variables and was presented using median with the 25^th^ to 75^th^ percentile interval, and categorical data were summarized as frequency and percentages. The bivariate analyses were done using the Mann-Whitney U test, and for categorical variables the Chi-square test or Fisher’s exact test according to the nature and distribution of the variable. Statistical significance was established at alpha level of 0.05. All statistical analyses were performed using IBM Corp. Released 2021, IBM SPSS Statistics for Windows, Version 28.0, Armonk, NY: IBM Corp. and STATA 15 (Stata Corp. 2017. Stata Statistical Software: Release 15. College Station, TX: Stata Corp LLC) for Windows.

## RESULTS

A total of 107 patients diagnosed with ATAAD were identified, 69 underwent hemiarch repair, and 38 underwent TAR (26 FET), as shown in Figure 1. Patient’s baseline characteristics are displayed in Table 1.

**Table 1:** Preoperative baseline characteristics.

	Hemiarch n=69	TAR n=38	Total n=107	*P-* value
Age, years	58 (49-67)	56 (46-65)	57 (49-66)	0.36
Male	45 (65)	29 (73.3)	74 (69.2)	0.23
Diabetes	4 (5.8)	1 (2.6)	5 (4.7)	0.65
Dyslipidaemia	13 (18.8)	4 (10.5)	17 (15.9)	0.41
Hypertension	46 (66.7)	30 (79)	16 (71)	0.18
Chronic obstructive pulmonary disease	2 (2.9)	3 (7.9)	5 (4.7)	0.35
Stroke	4 (5.8)	3 (7.9)	7 (6.5)	0.70
Previous cardiac surgery	6 (8.7)	6(15.8)	12 (11.2)	0.27
NYHA				
I	8 (11.6)	5 (13.2)	13 (12.2)	1.00
II	45 (65.2)	30 (79)	32 (30)	0.14
III	6 (8.7)	2 (5.3)	8 (7.5)	0.71
IV	10 (14.5)	1 (2.6)	11 (10.3)	0.09
Left ventricular ejection fraction, %	51 (50-55)	51 (51-60)	51 (50-57)	0.26
Timing of dissection				0.45
Hyperacute (< 24 h)	4 (5.8)	4 (10.5)	8 (7.5)	
Acute (24 h - < 2 weeks)	65 (94.2)	34 (89.5)	99 (92.5)	
Euro SCORE II	7 (4-14)	7 (5-9)	7 (4-12)	0.74

EuroSCORE=European System for Cardiac Operative Risk Evaluation; NYHA=New York Heart Association; TAR=total arch replacement

Results are expressed in n (%) or median (interquartile range)

### Preoperative

Both groups had comparable preoperative characteristics. The most frequent comorbidity was hypertension, followed by dyslipidaemia in both groups. There were no differences in functional class between groups, and most patients had NYHA functional class II. The median left ventricular ejection fraction in both groups was 51%. Comparing the timing of dissection, 7.5% of the patients presented in the first 24 hours from the onset of symptoms, and 92.5%o consulted in a > 24 hours-14 days interval after the onset. There were no differences in the median preoperative mortality risk between the two groups.

### Intraoperative

**Fig. 2:**
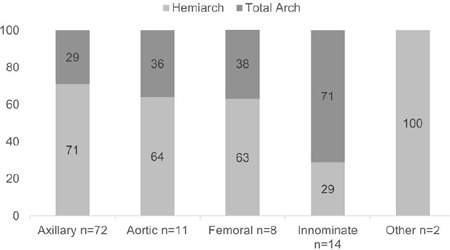
Connulotion sites’ percentages. TAR=total arch replacement.

Intraoperative results are displayed on Table 2. Most patients underwent surgery as an emergency surgical priority (n=78, 72.9%o), 26 (24.3%) as urgent, and three (2.8%) as emergent salvage. The mean duration of CPB was significantly longer (*P*=0.01 ) in TAR (242 min [210 min-290 min]) when compared to the hemiarch repair group (217 min [168 min-253 min]). The median aortic crossclamping time was similar for both groups (129 min *vs.* 132 min, *P*=0.83).The most frequent cannulation site was the axillary artery as shown in Figure 2. From the 38 patients who underwent TAR, 26 patients underwent surgery by hybrid approach with FET, and 12 cases by the conventional elephant trunk technique. Proximal extension of the repair included ascending aortic replacement in 57%o of patients, aortic root replacement in 38%o, Bentall procedure in 30%o, and valve-sparing aortic root replacement in 9%o of cases. Aortic valve interventions were repairs in 13% of cases, and replacements in 3%.

**Table 2:** Intraoperative characteristics.

	Hemiarch n=69	TAR n=38	Total n=107	*P-*value
Surgical priority				
Urgent	9 (13)	17 (44.7)	26 (24.3)	< 0.001
Emergency	58 (84.1)	20 (52.6)	78 (72.9)	< 0.001
Emergent salvage	2 (2.9)	1 (2.6)	3 (2.8)	1.00
Cardiopulmonary bypass time (min)	217 (168-253)	242 (210-290)	224 (186-264)	0.01
Aortic cross-clamping time (min)	132 (100-163)	129 (97-196)	132 (100-165)	0.83
Cerebral perfusion (min)	24 (20-31)	40.5 (30-54)	30 (22.5-41.5)	< 0.001
Lowest temperature (ºC)	25 (23-32)	24 (22-25)	24 (23-26)	0.13
Cannulation site				
Axillary	51 (73.9)	21 (55.3)	72 (67.3)	0.06
Aorta	7 (10.1)	4 (10.5)	11 (10.3)	1.00
Femoral	5 (7.3)	4 (10.5)	9 (8.4)	0.72
Innominate	4 (5.8)	10 (26.3)	14 (13.1)	0.01
Other	2 (2.9)	0	2 (1.9)	0.54
Associated procedures				
Aortic valve repair	8 (11.8)	6 (15.4)	14 (13.1)	0.90
Aortic valve replacement	2 (2.9)	1 (2.6)	3 (2.8)	0.90
Ascending aortic replacement	39 (57.4)	22 (56.4)	61 (57)	1.00
Aortic root replacement	28 (41.2)	13 (33.3)	41 (38.3)	0.54
Bentall procedure	23 (33.8)	9 (23.1)	32 (29.9)	0.28
Valve-sparing root replacement	5 (7.4)	4 (10.3)	9 (8.4)	0.72

TAR=total arch replacement

Results are expressed in n (%) or median (interquartile range)

### Postoperative

Results of postoperative outcomes are shown in Table 3. There were no significant differences in terms of mortality (*P*=0.45), stroke rate (*P*=0.06), and SCI. There were no differences in secondary postoperative outcomes such as surgical site infection or AKI. Bleeding reoperation was higher in the hemiarch group, but no statistically significant difference was found (*P*=0.05) when compared to the total arch group. There was a total of 18 (16.8%) in-hospital deaths, six (5.6%) cases of stroke, and two cases of SCI were identified. In-hospital mortality rate was 18.8% for the hemiarch repair group and 13.2%o for the total replacement group. Median total length of stay was significantly longer (*P*=0.03) in TAR (16 days [11 days-27 days]) than hemiarch repair (12 days [8 days-20 days]) with no significant differences in ICU stay.

**Table 3:** Postoperative outcomes.

	Hemiarch n-69	TAR n-38	Total n=107	*P-*value
Surgical site infection				
Sternal	1 (1.5)	1 (2.6)	2(1.9)	1.00
Mediastinal		1 (2.6)	1 (0.9)	0.36
Bleeding reoperation	21 (30.4)	4 (10.5)	25 (23.4)	0.05
Stroke	1 (1.5)	5 (13.2)	6 (5.6)	0.06
Acute kidney injury	10 (14.5)	5 (13.2)	15 (14)	1.00
Spinal cord injury, > 24 h				0.06
Transient lower extremity paresis		1 (2.6)	1 (0.9)	0.30
Permanent lower extremity paralysis		1 (2.6)	1 (0.9)	0.30
ln-hospital mortality	13 (18.8)	5 (13.2)	18 (16.8)	0.45
Hospital length of stay (days)				
ICU	6 (3.8-14)	7 (4-11)	6 (4-12.5)	0.90
Total	12 (8-20)	16 (11-27)	13 (9-24)	0.03

ICU=Intensive care unit; TAR=total arch replacement

Results are expressed in n (%) or median (interquartile range)

## DISCUSSION

ATAAD is a life-threatening emergency that requires immediate surgical treatment. The main goal of surgical repair for ATAAD is preventing death and its complications in the short term by the resection of the initial dissection tear. The management of ATAAD has been evolving throughout the years, however, there is still no consensus regarding the most adequate extension of the repair. The available evidence shows contradictory information about the early-, mid-, and long-term outcomes when comparing hemiarch repair *versus* TAR. Recent studies have started to show better long-term outcomes with an extended replacement in specific populations^[15]^.

Some series show better outcomes with the hemiarch repair. Kim J et al. reported a retrospective cohort study including 188 patients with DeBakey I aortic dissection that showed greater late death rate (*P*≤0.05) for the total arch group 22.7% (n=10) as compared to the hemiarch group 9.7% (n=14)^[16]^. Likewise, a systematic review and meta-analysis conducted by Ma et al.^[17]^ concluded that hemiarch replacement provides better early mortality rate, lower permanent neurological dysfunction, renal failure, and dialysis in this type of patients.

On the other hand, other contemporary series have reported no differences in terms of mortality rate with the TAR as the studies by Ok et al.^[18]^, Vendramin et al.^[19]^, and Hayashi et al.^[20]^ show. Patel et al.^[21]^ and Omura et al.^[22]^ also reported similar outcomes in terms of early mortality. Summarized data of the studies presented above are displayed on Table 4. A systematic review and metaanalysis conducted by Poon et al.^[23]^ including 14 retrospective cohort studies for a total of 2,221 patients reported no statistically significant differences of in-hospital mortality between hemiarch and TAR with a mortality rate of 3.60-24.1% and 3.85-28.57% (*P*=0.20), respectively.

**Table 4:** Summarized mortality data from other reports.

	Overall	TAR	Non-TAR	*P*-value
Omura et al.^[22]^, 30-day mortality	18 (9.1)	6 (6.8)	12 (11)	0.44
Omura et al.^[22]^, hospital mortality	25 (12.7)	9 (10.2)	16 (14.7)	0.47
Ok et al.^[18]^, early deaths	-	8 (6.8)	23 (9.3)	0.56
Ok et al.^[18]^, late death	-	26 (22.2)	68 (27.4)	0.35
Vendramin etal.^[19]^, era 1 30-day	-	3 (15)	13 (15)	1
Vendramin et al.^[19]^, era 2 30-day	-	1 (1)	6 (8)	0.24
Hayashi et al.^[20]^, in-hospital mortality	-	1 (1)	7 (4)	0.17
Hayashi et al.^[20]^, 30-day mortality	-	1 (1)	5 (2.8)	0.33
Hayashi et al.^[20]^, late death	-	2 (2)	34 (19)	< 0.01
Patel et al.^[21]^, early death	76 (12)	11 (10.3)	39 (9.9)	0.01*

TAR=total arch replacement

*Authors included the no arch intervention group (n=130) in the analysis which had 26 deaths (20%) Results are displayed in n (%)

Our study compared the outcomes of 107 patients diagnosed with ATAAD who underwent aortic repair surgery in a 20-year period. We reviewed the impact of the graft extension on major early outcomes. Even in high volume centres, mortality remains high, ranging from 17% to 26% ^[4,24]^. However, in coherence with the aforementioned studies, our results showed no significant difference in terms of early mortality, stroke rate, or SCI.

The hemiarch repair has been associated with an increased risk of residual dissection as it demands a longer suture line for distal anastomosis. Is has been well established that residual dissection in the early phase causes an unfavourable distal remodelling and a greater risk of complicated aortic dilatation^[25]^. Whereas a TAR performed by either conventional or FET technique provides a safer anastomosis and distal remodelling^[25]^, therefore providing a greater long-term survival and lesser risk of reoperation in patients with ATAAD^[15]^.

Even if the TAR is a more complex and technically demanding procedure, the surgical strategy for ATAAD has been migrating towards favouring extended repairs as these have begun to show improved long-term outcomes with comparable short- and mid-term results if performed in experienced centres. While in the early phase of our study we performed the hemiarch repair more frequently, with the appearance of newer techniques the number of extended, and hybrid repairs has been gradually increasing over time. Surgeons in our study preferred the FET technique over the classic elephant trunk forTAR.

ATAAD treatment is a technically challenging procedure. Thus, in some patients, it is still reasonabletolimittherepairtotheascending aorta. However, evidence seems to show that an extended arch replacement is preferred in the following situations^[26]^:

A. When the tear is located within the arch
B. In malperfusion syndrome
C. In younger patients
D. In patients with connective tissue disorders or hereditary aortic syndromes with an aortic aneurysm > 45 mm

Moreover, replacement with FET has shown to favour and facilitate distal aortic remodelling, future descending aortic surgery, and might reduce the number of future operations. However, performing an extended repair requires experience in aortic arch surgery^[27]^.

### Limitations

The limitations of our study are inherent to the retrospective observational design. Additionally, this was a single-centre experience in a referral centre in Colombia with a homogeneous and limited sample. Bias related to surgeon’s preference on the extension of the surgery was present as well because each surgeon had an approach based on patient’s condition and experience. Long-term follow-up was available in a very low percentage of patients, and hence long-term outcomes were not measured.

## CONCLUSION

ATAAD management remains as a challenge for cardiothoracic surgeons. Our results show thatTAR does not seem to increase the risk of major early postoperative complications when compared to hemiarch repair and it would even provide some benefits such as a lesser risk of reoperation. Therefore, we believe that TAR should be performed when feasible, in selected population, to improve long-term outcomes.

## Abbreviations, Acronyms & Symbols

AKI: Acute kidney injury
ATAAD: Acute type A aortic dissection
CPB: Cardiopulmonary bypass
EuroSCORE: European System for Cardiac Operative Risk Evaluation
FET: Frozen elephant trunk
ICU: Intensive care unit
NYHA: New York Heart Association
SCI: Spinal cord injury
STS: Society of Thoracic Surgeons
TAR: Total arch replacement
